# Exercise Promotes Bone Marrow Microenvironment by Inhibiting Adipsin in Diet-Induced Male Obese Mice

**DOI:** 10.3390/nu15010019

**Published:** 2022-12-21

**Authors:** Zunhan Shi, Lihui Wang, Jinwen Luan, Liqin Yin, Xiaohui Ji, Wenqian Zhang, Bingxiang Xu, Linshan Chen, Ying He, Ru Wang, Longhua Liu

**Affiliations:** 1 School of Exercise and Health, Shanghai University of Sport, Shanghai 200438, China; 2 Department of Medical Imaging, Shanghai East Hospital (East Hospital Affiliated to Tongji University), Tongji University, Shanghai 200123, China; 3 Department of Pathology and Cellular Biology and Naomi Berrie Diabetes Center, Columbia University, New York, NY 10027, USA

**Keywords:** exercise, obesity, adipsin, bone resorption, alternative complement pathway

## Abstract

Obesity is a growing global epidemic linked to many diseases, including diabetes, cardiovascular diseases, and musculoskeletal disorders. Exercise can improve bone density and decrease excess bone marrow adipose tissue (BMAT) in obese individuals. However, the mechanism of exercise regulating bone marrow microenvironment remains unclear. This study examines how exercise induces bone marrow remodeling in diet-induced obesity. We employed unbiased RNA-Seq to investigate the effect of exercise on the bone marrow of diet-induced obese male mice. Bone mesenchymal stem cells (BMSCs) were isolated to explore the regulatory effects of exercise in vitro. Our data demonstrated that exercise could slow down the progression of obesity and improve trabecular bone density. RNA-seq data revealed that exercise inhibited secreted phosphoprotein 1 (Spp1), which was shown to mediate bone resorption through mechanosensing mechanisms. Interactome analysis of Spp1 using the HINT database showed that Spp1 interacted with the adipokine adipsin. Moreover, exercise decreased BMAT, which induced osteoclast differentiation and promoted bone loss. Our study reveals that exercise improves the bone marrow microenvironment by at least partially inhibiting the adipsin–Spp1 signaling pathway so as to inhibit the alternative complement system from activating osteoclasts in diet-induced obese mice.

## 1. Introduction

Characterized by excess adiposity and adipose tissue dysfunction, obesity has risen as an international public issue and often triggers many metabolic complications such as diabetes, cardiovascular diseases, and osteoporosis [1]. Osteoporosis is characterized by low bone mass and an elevated risk of bone fracture. Imbalanced bone homeostasis is the most fundamental cause of osteoporosis. In obese and aged diabetic patients, the cortical bone was surrounded by redundant marrow adipose tissue and increased cortical porosity. The bone homeostasis may be disrupted by the excess bone marrow adipose tissue (BMAT) in the bone cavity accompanied by these diseases [2,3]. Except as an energy depot, adipose tissue also regulates the whole body’s metabolic homeostasis. Distinct from white adipose tissues and brown adipose tissues, BMAT is located in the bone marrow cavity. It has been shown that BMAT regulates bone mass, hematopoietic function, metabolic homeostasis, and bone remodeling [4]. Aging and several diseases increase BMAT volume, including diabetes, obesity, and anorexia nervosa [5]. Increased BMAT is frequently associated with bone loss and worsens the condition. However, the mechanism by which this happens is still unclear. The skeleton is a dynamic organ composed of different cells including osteoblasts, bone marrow adipocytes, chondrocytes, etc., all of which are derived from bone mesenchymal stem cells (BMSCs) [6]. There is widespread support for the theory that when adipogenic and osteogenesis differentiation of BMSCs is in homeostasis, the bone marrow can perform its function normally. Studies have shown that bone marrow functions such as hematopoiesis depend on the homeostasis of BMAT and bone [7]. The current findings seem to indicate that obesity stimulates bone marrow adipogenesis and causes a deleterious effect on bone in later life [8].

Physical exercise, calorie control, and a healthy lifestyle are well-known ways to lose weight [9]. While regular calorie restriction (CR) does reduce body weight (BW), some adverse side effects of CR impede it from widespread use, such as perpetual hunger, cold feeling, and skeletal fragility including bone loss and increased BMAT [10]. Exercise has been proven to decrease BMAT volume and improve bone quality in high-fat diet–induced mice [11]. As a mechanical stimulation, exercise regulated the balance of bone and marrow fat mainly by inhibiting the adipogenic differentiation of BMSCs [12]. Among many factors regulating the differentiation process of BMSCs, PPARγ is a crucial factor that can promote bone marrow adipose tissue differentiation and suppress osteogenesis [13,14]. The activity of PPARγ is well regulated in different ways, including acetylation [15]. It has been shown that deacetylation of PPARγ on K268 and K293 prevented TZD-induced bone loss and increased insulin sensitivity [16,17,18].

Adipsin, one of the adipokines regulated by PPARγ, is decreased by deacetylation of PPARγ. Knockout of adipsin prevented CR-induced bone loss and increased osteoblast differentiation [19]. Furthermore, except as an adipokine, adipsin is a rate-limiting enzyme in the alternative complement pathway [20]. The complement pathway is a member of the innate immune system that defends against invading pathogens relying on activation of the classical, alternative, and lectin pathways [21]. As the central component of the complement pathway, protein C3 is the point of intersection in three distinct pathways. C3 spontaneous hydrolysis triggers the alternative complement pathway, and then the hydrolyzed C3 bonds with factor B to form a complex [22]. The success of the subsequent reaction is dependent on the cleavage of factor B by the adipsin [20]. It was shown that Adipsin played an important catalytic role in the formation of C3 invertase and the downstream activation of this pathway [23].

The previous study also showed that bone resorption is a prerequisite for bone formation [24]. Only functional osteoclasts can be anchored at the site of bone resorption for the following step. Osteoclasts attach to the bone surface through the interaction of cell surface αvβ3 integrins with secreted phosphoprotein 1 (Spp1) [25]. As a secreted glycoprotein, Spp1 is characterized as a structural matrix cellular protein which was shown to play a vital regulatory role in osteoclast activity and osteoclast number [25]. Meanwhile, it was shown that osteoclast differentiation was impaired in the bone marrow of adipsin knockout mice [26]. We also found a possible protein interaction between Spp1 and adipsin through the HINT database [27]. This database summarizes the contents of high-quality protein–protein interactions from eight interactome resources. Moreover, the HINT database is updated regularly and tracks all versions.

Unlike CR, exercise can restrain BMAT accumulated from obesity and increase bone density by bone remodeling through employing cell mechanosensitivity [28]. However, the mechanism of exercise-induced bone remodeling remains unknown. In this study, we aim to investigate how exercise induces bone remodeling under diet-induced obesity in a comprehensive way. We employed unbiased RNA-Seq to comprehensively investigate the exercise’s effect on the bone marrow of diet-induced obese mice in vivo. Further, BMSCs were isolated to explore the regulatory effects of exercise in vitro. Our data indicated that exercise improved the bone marrow microenvironment via mechanosensing mechanisms in obese mice by at least partially inhibiting the adipsin–Spp1 pathway. These findings are likely to provide new strategies for treating obesity and diabetes with fewer side effects on bone health.

## 2. Materials and Methods

### 2.1. Animal Studies

Eight-week-old male C57/B6 mice were purchased from the Model Animal Research Center. The mice were housed at room temperature (RT, 23.1° ± 1°) on a 12 h light/dark cycle, with free access to drinking water and eating a regular diet. After the acclimation period, the mice were provided an HFD (60% calories from fat, 20% from protein, and 20% from carbohydrates) for 6 weeks to induce obesity. Then the obese animals were randomly divided into 2 groups: the obesity group (*N* = 9) and the obesity-combined-with-exercise group (*N* = 9). The sample size was determined by power analysis using preliminary data obtained in our laboratory with the following assumptions: α of 0.05 (two-tailed), power of 0.8. We calculated the effect size based on experimental grouping and the statistical methods used. Then we calculated the sample size according to G * power, effect size d = 1.6, α = 0.05, 1 − β = 0.8, and calculated the required sample size to be 16 (control = 8, experimental group = 8). In the study, we used 9 mice per group. The exercise group ran 1 h of moderate-intensity exercise 6 days per week and maintained for 12 weeks. The obesity group had no interventions except for feeding HFD. Body composition was measured weekly by echo magnetic resonance imaging (MRI). For the insulin tolerance test (ITT), the mice were fasted for 4 h and then were injected with insulin (0.75 u/kg) at the beginning of the twelfth week. For the glucose tolerance test (GTT), the mice were fasted overnight and then injected with glucose (20%, 10 µL/kg). Blood glucose was measured at particular time points. We employed the TSE PhenoMaster system to monitor the mice’s basal metabolism before collecting the samples. All the animal protocols used were approved by the Ethics Review Committee for Animal Experimentation of the Shanghai University of Sport.

### 2.2. Bone Processing and Analysis

The femurs were separated and soaked in 4% formalin for at least 48 h, and subsequently used for scanning by micro-computed tomography (CT) and further bone density quantification using the Scanco viva CT 80 at a voxel size of 9 μm with an energy of 55 kV and 145 μA. Cortical and trabecular bone structures near the growth plate of the distal femur were reconstructed after the scan. We chose to reconstruct 1 mm centripetal from the growth plate and quantified cortical and trabecular bone volume by Scanco viva CT 80.

### 2.3. cDNA Library Preparation

Bone marrow was collected from our mice by centrifugation to make RNA-seq. The kit used to build the libraries was NEBNext^®^ Ultra™ RNA Library Prep Kit for Illumina^®^ (Ipswich, MA, USA). The input of the libraries was total RNA samples, and the total amounts and integrity were detected using an Agilent 2100 bioanalyzer. Meanwhile, the insert size of the cDNA libraries later formed was assessed by this approach. The mRNA with poly-A tail was enriched by Oligo (dT) magnetic beads, and then double-stranded cDNA was synthesized using the randomly fragmented mRNA as a template. The libraries were finally obtained after the process of end repair, adaptor ligation, and PCR amplification. The libraries were diluted to 1.5 ng/uL, and the effective concentration of the libraries was higher than 2 nM (qRT-PCR quantification).

### 2.4. RNA-Sequencing

After the library was qualified, the different libraries were pooled according to the effective concentration and the target amount of data from the machine, then sequenced by the Illumina NovaSeq 6000. The ending reading of 150 bp pairing was generated. About 51.55 million pair of reads with high sequencing quality were obtained for each library.

### 2.5. RNA-Seq Data Analysis and Data Visualization

The quality of all the libraries was assessed using FastQC (http://www.bioinformatics.babraham.ac.uk/projects/fastqc/, accessed on 25 November 2021). Reads with poor sequencing qualities, together with the adapter sequences, were then removed using fastp [29] with default configurations. The reads from the rRNA species were removed by filtering reads with mappability to the rRNA sequences. The cleaned reads were then mapped to the mouse genome GRCm38 using HiSAT2 (http://daehwankimlab.github.io/hisat2/, accessed on 4 March 2022). Coordinately aligned reads were filtered out using SAMtools [30]. Gene expression levels were quantified using the stringtie [31] with Gencode vM25 annotation together with read counts in each gene. Differential expression analysis was then performed using a DESeq2 package [32]. Genes with *p* value < 0.05 and |log_2_FoldChange| > 1 were determined as differentially expressed. GO and KEGG enrichment analysis of differentially expressed genes was based on DAVID (https://david.ncifcrf.gov/, accessed on 1 April 2022). Terms with a *p* value less than 0.05 were determined as significant. Gene Set Enrichment Analysis was implemented by the clusterProfiler R package (v 4.2.2, Guangchuang Yu, Guangzhou, China) [33].

### 2.6. BMSC Isolation

The mice were euthanized, and the tibias were separated. The BMSCs were isolated from the femurs employing a 10-gauge needle into MesenCult™ (Bucharest, Romania) Expansion Kit (Mouse) Catalog #05513 medium supplemented with 1% L-Glutamine. Reaching 70–80% confluence, the BMSCs were passaged and plated in stretcher plates for further experiments.

### 2.7. BMSC Culture and Treatment

We isolated the BMSCs from the mouse tibia and cultured the cells with a basal medium. Second- or third-generation mesenchymal stem cells were collected and incubated with an adipogenic differentiation medium containing 0.5 mM IBMX, 1μM dexamethasone, 1 μg/mL insulin, and 1 μM rosiglitazone with a high glucose medium in the 6-well culture plates. The bottom of these plates contained an elastic silicone membrane. Meanwhile, we employed the Flexcell FX-5000 Tension system to tension the membrane that imitated the exercise state. All the cells were divided into a control group and a stretch group; the control group’s culture conditions were consistent except for the stretch. The stretch parameters were as follows: 5% strain magnitude, frequency of 0.5 hz, 6 h/day [34]. We collected the cells to further analyze the mature lipid drops.

### 2.8. Quantitative Real-Time PCR

The total RNA was isolated from the femur using an Omega total RNA kit (R6812, HP total RNA kit), and the concentration and purity were determined by measuring the absorbance at 260 and 280 nm with a spectrophotometer. One ug RNA was reverse transcribed into complementary cDNA using the PrimeScript RT Reagent Kit (Takara, RR036A, Goteborg, Sweden). Amplification was carried out using a SYBR Premix Ex Taq (Takara, RR420A) and performed on an Applied Biosystems 7500 real-time PCR instrument. The PCR products were normalized to GAPDH, and the relative gene expression levels were calculated using the ΔΔCT method.

### 2.9. Western Blotting

The cells were lysed and the tissues were homogenized using a homogenizer (High Efficient Scientz-48 Tissue Grinder) after dissection in Western extraction buffer (Beyotime, P0013, Shanghai, China). The lysate was incubated on ice for 30 min and debris was removed by centrifugation. SDS-PAGE and western blotting were performed and detected with ECL (Millipore, Burlington, MA, USA, WBKLS0100). These antibodies included anti-adipsin (Santa Cruz Biotechnology, Dallas, TX, USA, catalog#47683) and anti-beta actin (Santa Cruz Biotechnology, catalog#47778).

### 2.10. Statistics

Significant differences were determined using the Student *t*-test. * *p* < 0.05 was considered to be a significant difference. All the data were analyzed using Graphpad Prism9 software, (9.0.0 121, Dr. Harvey Motulsky, San Diego, CA, USA) and the results are expressed as mean ± standard deviation.

## 3. Results

### 3.1. Exercise Alleviated Diet-Induced Insulin Resistance and Improved Trabecular Bone Density

**Figure 1:**
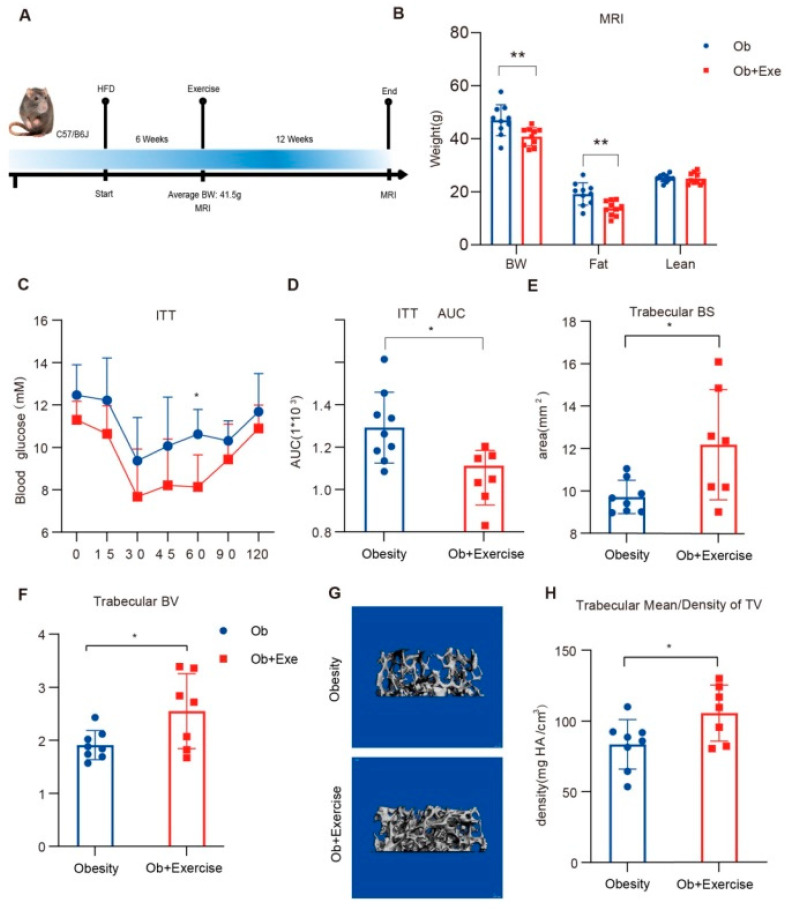
Exercise improved insulin sensitivity and bone density. (**A**) The eight-week-old male C57/B6 mice were fed with HFD for 6 weeks. The average body weight of the mice achieved 41.5 g after 6 weeks of HFD and before the mice started the 12-week treadmill running with HFD. Body composition was measured before and after exercise intervention. (**B**) Body composition was measured after exercise in the obesity and exercise groups using MRI. (**C**,**D**) measurement of plasma glucose during ITT and the AUC of the obesity (*N* = 9) and exercise (*N* = 7) groups after 8-week treadmill exercise. (**E**,**F**) femoral BS, BV in the trabecular regions of TV. (**G**) representative trabecular reconstruction image of the femur by μCT. The gray part of the picture shows reconstructed trabecular bones. (**H**) femoral bone density in the trabecular regions of TV. * *p* < 0.05, ** *p* < 0.01 2-sided Student *t* test. Data are presented the mean ± SD. AUC, areas under the curve; BW, body weight; HFD, high fat diet; ITT, insulin tolerance test; MRI, magnetic resonance imaging.

To investigate the potential mechanism of exercise-induced bone remodeling in obese conditions, eight-week-old male C57/B6 mice were fed with HFD to establish an obese mice model. Then the obese mice were randomly divided into two groups—the obesity group (Ob) and the obesity-combined-with-exercise group (Ob + Exe)—with no significant differences in body weight, fat mass, or lean mass (Supplementary Figure S1A). The exercise group was put on a moderate-intensity treadmill running for 12 weeks (Figure 1A). Body weight data indicated that exercise could postpone excessive weight gain even under an HFD. The body weight of the mice in the exercise group was less than in the obesity group (Figure 1B), accompanied by significantly reduced HFD-induced fat expansion but not lean mass (Figure 1B). These data showed that exercise reduced diet-induced fat expansion. Since obesity is a high-risk factor for diabetes, we tested whether exercise could improve insulin sensitivity in obese mice. Our plasma ITT result indicated that exercise could improve the insulin sensitivity of obese mice (Figure 1C,D), with no significant difference in GTT (Supplementary Figure S1B), which indicated that exercise improved the insulin sensitivity of insulin-targeted metabolic tissues (such as adipose tissue) rather than affecting the function of pancreatic β cells. Obesity-related osteoporosis is always accompanied by bone loss, while exercise might increase bone density. From the micro-CT quantitative analysis, bone density was enhanced during exercise, especially in the trabecular bone (Figure 1E–H). Trabecular bone area, volume, and mean density improved significantly after 12 weeks of moderate-intensity exercise in the obese mice, which was consistent with previous studies [35]. The trabecular area and density increased by 28% and 26%, respectively. In our obese mouse model, 12 weeks of moderate-intensity exercise improved diet-induced insulin resistance and bone density, especially in the trabecular bone.

### 3.2. Exercise Changed the Bone Marrow Microenvironment via Mechanosensing Mechanisms

**Figure 2:**
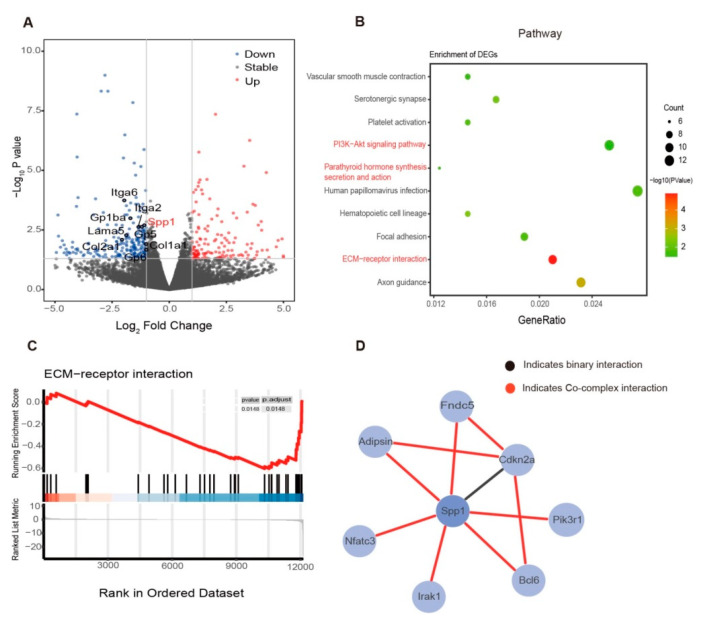
RNA-Seq of bone marrow indicated that exercise inhibited Spp1 and related ECM-receptor pathways. Representative bone marrow from tibia of both the obesity and exercise groups (*N* = 2,2) were isolated for RNA-Seq analysis. (**A**) volcano plot of bone marrow in the exercise group compared to the obesity group. Colored dots represent *p* value < 0.05 (−log_10_(*p* Value) > 1.3, gray line), log_2_Fold Change > 1 up-regulated genes (red), and log_2_Fold Change < −1 down-regulated genes (blue). Selected ECM-receptor pathway related genes are highlighted. (**B**) results of pathway enrichment analysis for highly variable genes identified by significant DEG in the bone marrow. (**C**) gene set enrichment analysis of ECM-receptor interaction. (**D**) protein interaction network diagram of Spp1. The original data come from the HINT (http://hint.yulab.org, accessed on 31 May 2022) database, a database of high-quality protein–protein interactomes. ECM, extracellular matrix.

To investigate how exercise induces bone remodeling in diet-induced obesity in a comprehensive way, we isolated the bone marrow from the tibia after the 12-week exercise intervention in the control group and analyzed the organs by RNA-seq (Ob vs. Ob + Exe). Out of 12,028 differentially expressed genes (Figure 2A), KEGG pathway analysis showed that the canonical pathway that was significantly inhibited was the ECM-receptor interaction pathway related to tissue function and structure, cell motility, proliferation, and differentiation (Figure 2B). Gene set enrichment analysis also confirmed this finding (Figure 2C). The mechanical environment consists of cell–cell and cell–ECM interactions. Extracellular matrix (ECM) provides a scaffold for intercellular biochemical or biomechanical communication. Focal adhesions were mechanosensory complexes which also showed up in our pathway enrichment results (Figure 2B). Integrins and perhaps other cell surface–associated components mainly mediate the interactions between cells and the mechanical environment. This interaction affected cellular activities and impacted the mesenchymal progenitor cell (MPC) fate [36]. We also found that the Spp1, which has been implicated as an essential factor in bone remodeling, was involved in the ECM-receptor interactions, in focal adhesion, and in the PI3K-Akt signaling pathway (Figure 2A,B). Meanwhile, Spp1 is necessary for osteoclasts’ function via interacting with the integrin αvβ3 [37]. To further study how exercise regulates Spp1, an HINT database was used to search the interacted proteins for Spp1. Adipsin, an adipokine, caught our attention through protein interaction analysis of Spp1 by means of the HINT database [27] (Figure 2D). Adipsin is secreted by adipose tissue and participates in the complement system alternative pathway. The protein interaction between Spp1 and adipsin has been confirmed [38]. We wonder whether adipsin mediated the bone and BMAT change in the obese mice during exercise. Mechanical signals transmitted through the mechanical environment often affect cell behavior and phenotype [39]. Our RNA-seq results revealed that exercise might improve bone health by altering BMAT via mechanosensing mechanisms.

### 3.3. Exercise Decreased Bone Marrow Spp1 and Adipsin Level While Inhibiting Bone Resorption

**Figure 3:**
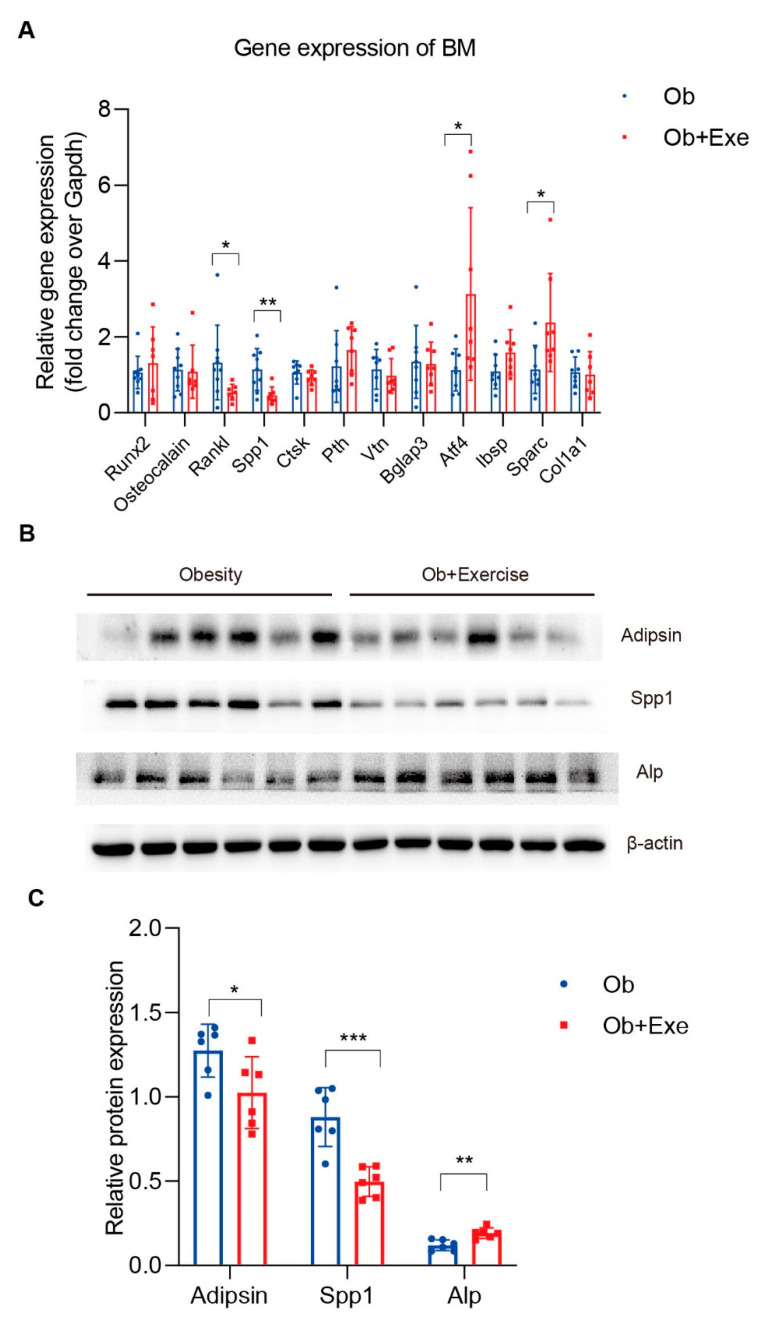
Exercise inhibited bone resorption and protected bone by reducing adipsin and Spp1. Quantitative real-time PCR analyses and protein expression level of tibia BM. (**A**) Bone remodeling related gene expression quantitative analysis of bone marrow. The bone marrow was isolated from the male C57/B6 mice (*N* = 9) that were induced for 6 weeks of high-fat diet and intervened in twelve weeks of exercise. * *p* < 0.05, ** *p* < 0.01, *** *p* < 0.001 for obesity group (*N* = 9) vs exercise group (*N* = 7). (**B**,**C**) Western blotting and quantification (*N* = 6) in tibia bone marrow from obesity group and exercise group. * *p* < 0.05, for obesity group (*N* = 6) vs exercise group (*N* = 6). * *p* < 0.05, ** *p* < 0.01 2-sided Student *t* tests were used for statistical analyses. Data are presented as mean ± SD. BMAT, bone marrow adipose tissue; PCR, polymerase chain reaction.

To further investigate the potential mechanism of exercise-induced bone remodeling, we isolated tibia bone marrow from both the obesity and exercise groups and examined the gene expression of some representative genes involved in bone remodeling (Figure 3A). It is well-known that Runx2 is responsible for inducing the differentiation of multipotent mesenchymal cells into immature osteoblasts [40]. In our obese mice model, exercise had no impact on the gene expression of Runx2 (Figure 3A). However, our results showed that Atf4, the regulator of osteoblast differentiation, was significantly upregulated after exercise (Figure 3A). Meanwhile, we also found that the expression of Alp protein, a marker of osteoblast differentiation maturation, was upregulated after exercise (Figure 3B,C). We evaluated critical factors involved in bone resorption, such as Rankl and Spp1. Consistent with our RNA-seq results, Spp1 was reduced by 60% by exercise at gene levels. In addition, we found that Spp1 protein levels were also significantly down-regulated, which means that osteoclasts cannot be anchored to the bone surface and thus interrupt the bone resorption process (Figure 3A–C). Rankl, another critical factor for osteoclast function, mediates bone resorption and overall bone density. A decline of it in the process of exercise could reduce osteoclast activity [41] (Figure 3A). These data strongly indicated that exercise could increase bone density in obese mice by inhibiting bone resorption. Meanwhile, other studies have shown the reduction in BMAT by exercise [35]; we wonder about the role of BMAT played in bone resorption. As the executor of bone resorption, osteoclast is modulated by several hormones, the immune system, and osteoblasts. Adipsin is an adipokine that in humans is encoded by the CFD gene, and it is mainly synthesized and secreted by adipocytes. It has been shown that adipsin can promote bone marrow adiposity [19]. In addition, adipsin, as a rate-limited enzyme involved in the alternative complement pathway, is required for efficient osteoclast differentiation [20,21,26]. Therefore, we asked whether exercise could decrease adipsin so as to inhibit bone resorption. Indeed, adipsin was significantly reduced 19.6% in the bone marrow by means of exercise compared to the obesity group (Figure 3B,C).

### 3.4. Mechanical Stretch Restrained BMSCs’ Adipogenic Differentiation and Suppressed Adipsin

**Figure 4:**
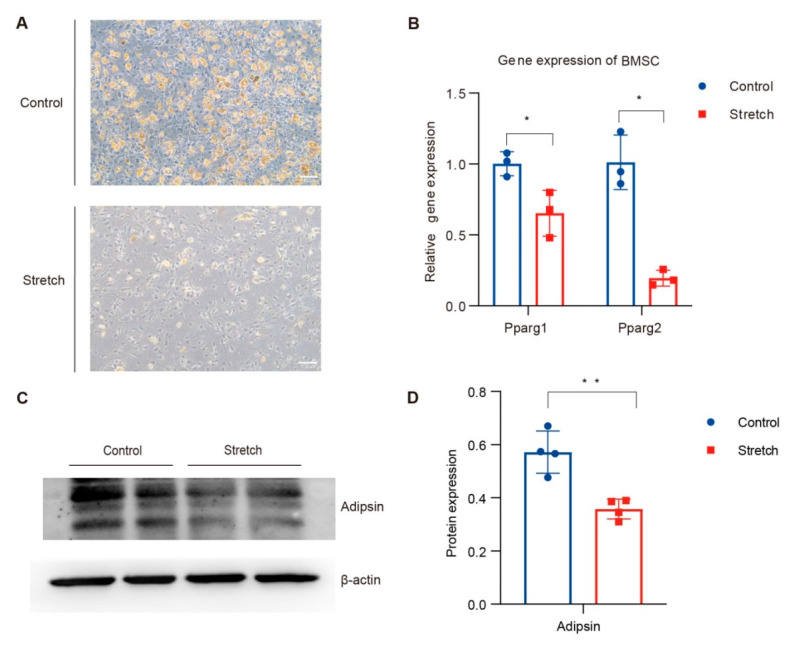
Exercise inhibited adipogenic differentiation of BMSCs and decreased adipsin in vitro. Isolated tibia mesenchymal stem cells were divided into control group and stretch group during adipogenic differentiation (*N* = 3). (**A**) representative images of control and stretch groups under a 10× phase-contrast microscope at the end of adipogenic differentiation. (**B**) quantitative PCR analysis of adipogenic relative gene expression in the BMSCs, containing Pparg1 and Pparg2. (**C**,**D**) representative Western blotting images and protein expression quantification (*N* = 4) of adipsin from mesenchymal stem cells treated with adipogenic differentiation and stretch stimulation. * *p* < 0.05, ** *p* < 0.01 for control group vs stretch group. Two-sided Student t-tests were used for statistical analyses. Data are presented as the mean ± SD. BMSCs, bone mesenchymal stem cells.

To verify the effect of exercise on bone marrow adipocyte and its adipokines, adipsin and BMSCs were isolated from the tibia, and then we induced adipocyte differentiation based on the well-established method with or without stretch by Flexcell FX5000 tension system to mimic the exercise or control groups in vitro [19]. The stretching stimulation of the mimic exercise inhibited lipid production and mature lipid droplet generation of BMSCs (Figure 4A). QPCR data also demonstrated that the mechanical stretch of BMSCs inhibited their adipocyte differentiation indicated by the significant decrease in important factors such as Pparg1 and Pparg2 (Figure 4B). Then we analyzed whether mechanical stretch affected the level of adipsin in BMSC-differentiated adipocytes. As expected, the protein level of adipsin reduced by 37.4% in the stretch group compared to the control group (Figure 4C,D). Therefore, exercise-mimicked mechanical stretch inhibited the adipocyte differentiation of BMSCs and reduced their adipokine adipsin.

## 4. Discussion

**Figure 5:**
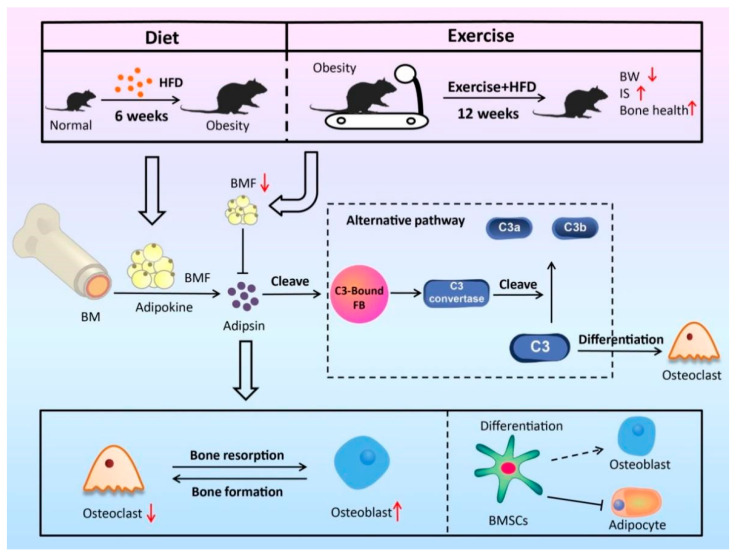
Exercise inhibited adipsin-mediated complement pathway activation on osteoclasts. Adipsin plays an essential role in C3 convertase forming and activation of the subsequent alternative complement pathway. As the central component of the complement pathway, C3 and the complement pathway are important in osteoclast differentiation. Exercise inhibits the adipokine adipsin expression and affects osteoclasts through its mediated complement system. In addition, exercise can restrain BMSC adipogenic differentiation to protect bone health. IS, insulin sensitivity; BW, body weight.

Here, we comprehensively investigated how exercise affected the bone marrow microenvironment in diet-induced obese male mice in vivo using unbiased RNA-Seq. BMSCs were isolated to explore the regulatory effects of exercise in vitro. Exercise delayed HFD-induced obesity and increased bone mass. Our data indicated that exercise improved the bone marrow microenvironment via mechanosensing mechanisms in obese mice by at least partially inhibiting the adipsin–Spp1 pathway, which indicated that bone marrow adipose tissue plays an active role in regulating bone marrow microenvironment by secreting adipokines, such as adipsin (Figure 5).

Twelve weeks of treadmill training improved insulin sensitivity compared to the obesity group (Figure 1C,D). Adipose tissue was a filler in the bone marrow cavity and was deemed to participate in local or even systemic energy metabolism. It also played a regulatory role that affected osteogenesis and hematopoiesis by paracrine and endocrine functions [42]. Exercise has been proven to alleviate diet-induced BMAT accumulation and improve bone health [11]. Adipocytes and osteoblasts of bone marrow shared the same progenitor cell, BMSCs [43]. Mesenchymal stem cell has has a great capacity for self-renewal while maintaining its multipotency, and its fateful differentiation selection depends on the dynamic balance in healthy individuals. There is a widespread hypothesis supporting the theory that BMSCs differentiate into a single lineage by the manner of competing with each other [44]. Interestingly, mature adipocytes and osteoblasts from human bone marrow were proven to be capable of transdifferentiation in vitro [45]. Moreover, the new technology lineage tracing system showed that the progenitors might already be committed to one lineage. Our study has shown that exercise inhibited BMSCs adipogenic differentiation. However, whether exercise could reverse the adipocytes and osteoclasts of bone marrow in vitro and the effects of exercise on bone marrow progenitors is unclear.

According to the observations of physiological conditions, the expansion of BMAT may proceed more extensively than bone mass alteration. Even though it differs from several other types of adipose tissue, the BMAT as an endocrine organ can secret adipokines and inflammatory factors to affect local or system metabolic processes. Prx1-Cre; Lepr(^fl^/^fl^) mice exhibited increased osteogenesis accompanied by decreased adipogenesis [46]. Adiponectin-KO (another important adipokine) mice were observed to have decreased whole-skeleton BMD [47]. Here, we firstly indicated that exercise increased bone density in obese mice by at least partially inhibiting adipokine adipsin. Bone forming is always followed by bone resorption, and these two processes of bone remodeling are coupled both spatially and temporally. In addition, the BM cells-secreted complement pathway component, such as C3, is required for efficient osteoclast differentiation [26]. Adipsin plays an essential role in the activation of C3 convertase and downstream pathway functioning [48]. In addition, our RNA-seq data showed that exercise decreased the coagulation-related biological process (Figure 2B and Supplement Figure S1C). Coagulation and complement activation are interdependent and mutually amplified [49]. Our RNA-seq data were also enriched to mediate a significant decrease in Spp1 that mediates the attachment of osteoclasts to the bone surface. The protein–protein interaction data of adipsin and Spp1 from the HINT database provide new insights for us. We reasonably speculate that exercise improves bone health in obese mice and is possibly achieved by inhibiting osteoclast-dominated bone resorption by the adipsin–Spp1 pathway. Compared to adipsin knockout mice, exercise downregulation of adipsin may have little effect on the activation and differentiation of osteoclasts. However, for mature osteoclasts during bone resorption, the function is equally important. Bone marrow is the primary site of hematopoietic stem cell (HSC) maintenance and hematopoiesis. Osteoblast has been shown to regulate hematopoietic stem cell function through Notch activation [50]. Interestingly, exercise decreased the hematopoietic cell lineage- and coagulation-related pathway in our study. We speculate that exercise can alleviate the disorder of the bone marrow microenvironment’s cellular components caused by obese-induced adiposity.

Fate decision of BMSCs is important for the bone marrow microenvironment. Adipsin has been shown to reduce the Wnt3a-induced phosphorylation of GSK3β and to promote adipose tissue production [19]. Our study found that adipsin levels decreased by means of exercise in both in vivo and in vitro models. Based on the above evidence, we speculated that exercise promotes the differentiation of mesenchymal stem cells into osteoblasts by inhibiting the expression of adipsin in the bone marrow. Osteoclast was shown to mediate the osteoblast precursors’ differentiation and proliferation in the bone marrow [11]. We suggested that employing adipsin on precursors or mature osteoclasts and osteoblasts in vitro may uncover the mechanism between alternative complement pathway and bone remodeling. Meanwhile, we reasonably speculate that the ECM-receptor interaction pathway in which Spp1 participates possibly regulates bone metabolism by affecting the function of osteoclasts. The protein interaction between adipsin and Spp1 may provide new ideas for exercise to regulate the dynamic changes in bone marrow adipose tissue and bone.

## Data Availability

All RNA-seq dataset is publicly available (GSE205676) at the GEO (https://www.ncbi.nlm.nih.gov/geo/info/linking.html). Accessed on 8 June 2022.

## Supplementary Materials

The following supporting information can be downloaded at: https://www.mdpi.com/article/10.3390/nu15010019/s1, Figure S1: Eight-week-old male C57/B6 mice were fed with HFD for 6 weeks to establish an obese mice model, and then these obese male C57/B6 mice were divided into two groups randomly: The obesity group (Ob) and the exercise group (Ob + Exe) (*N* = 9,9). (A) Body composition was measured before exercise for these two groups using MRI. (B) Measurement of plasma glucose during GTT of the obesity (*N* = 9) and exercise (*N* = 7) group. (C) Representative bone marrow from tibia of both the obesity and exercise groups (*N* = 2,2) was isolated for RNA-Seq analysis. Results of biological process analysis for highly variable genes identified by significant DEG in the bone marrow. Two-sided Student t-tests were used for statistical analyses. Data are presented as mean ± SD. BW, body weight; GTT, Glucose tolerance test; MRI, magnetic resonance imaging
