# Supplementary material for: Exercise Promotes Bone Marrow Microenvironment by Inhibiting Adipsin in Diet-Induced Male Obese Mice
Source: Nutrients. 2022 Dec 21;15(1):19. doi: 10.3390/nu15010019 (PMC9823335; doi:10.3390/nu15010019)
Supplement: Supplementary file 1 [file nutrients-15-00019-s001.zip › nutrients-1964573-supplementary.pdf]

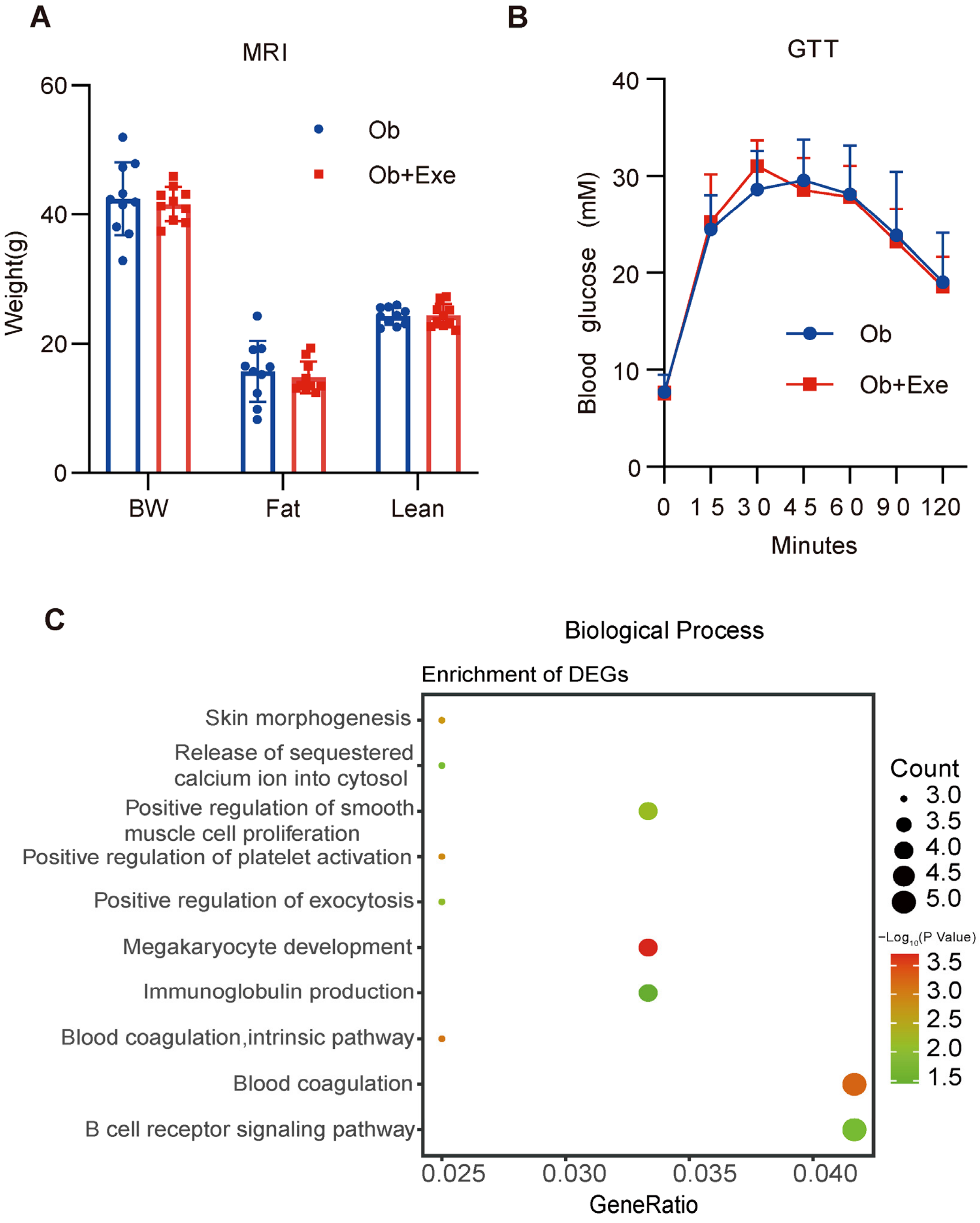

Figure S1: Eight-week-old male C57/B6 mice were fed with HFD for 6 weeks to establish an obese mice model, and then these obese male C57/B6 mice were divided into two groups randomly: The obesity group (Ob) and the exercise group (Ob + Exe) ($N = 9,9$). (A) Body composition was measured before exercise for these two groups using MRI. (B) Measurement of plasma glucose during GTT of the obesity ($N = 9$) and exercise ($N = 7$) group. (C) Representative bone marrow from tibia of both the obesity and exercise groups ($N = 2,2$) was isolated for RNA-Seq analysis. Results of biological process analysis for highly variable genes identified by significant DEG in the bone marrow. Two-sided Student t-tests were used for statistical analyses. Data are presented as mean ± SD. BW, body weight; GTT, Glucose tolerance test; MRI, magnetic resonance imaging
